# Women's Footprint in Anti-Doping Sciences: A Bibliometric Approach to Research Impact

**DOI:** 10.3389/fspor.2022.866648

**Published:** 2022-05-30

**Authors:** Anna Kiss, Zoltán Lakner, Sándor Soós, Andrea Petróczi

**Affiliations:** ^1^Library and Information Centre, Department of Scientometrics and Science Policy, Hungarian Academy of Sciences, Budapest, Hungary; ^2^Faculty of Education and Psychology, Eötvös Loránd University (ELTE), Budapest, Hungary; ^3^Department of Agricultural Business and Economics, Institute of Agricultural and Food Economics, Hungarian University of Agriculture and Life Sciences, Budapest, Hungary; ^4^Faculty of Agriculture, Kebbi State University of Science and Technology, Aliero, Nigeria; ^5^School of Life Sciences, Pharmacy and Chemistry, Kingston University, London, United Kingdom; ^6^Department of Movement Sciences, KU Leuven, Leuven, Belgium; ^7^Willibald Gebhardt Institute, University of Münster, Münster, Germany

**Keywords:** anti-doping, bibliometric mapping, gender equality, research impact, sport

## Abstract

Bibliometrics, *via* the exploitation of large-scale publication data, is a facile approach to explore gender-related trends, especially gender equality in academic publishing and authorship. For the first time, this study aims to investigate the gender-related trends in anti-doping sciences to (1) explore the relational structure of gender aspects of authorial, topical, and methodological features, (2) give recognition to women's contribution to anti-doping research, and (3) identify unique “gendered” potentials for advancing anti-doping research. To deliver on these aims, we employed bibliometric tools to publication records in anti-doping. After constructing a database containing academic publications on any aspect of anti-doping with at least one woman among the authors, we applied state-of-the-art methods from bibliometric science mapping and network analysis. The Lotka distribution model showed that the anti-doping research is a closed community with only 70 authors appearing more than once. Male authors being the majority (66.2%), women are under-represented in this field. The most important authorship position in the academic articles is mainly occupied by men, publications with male corresponding authors were in 774 out of 991 anti-doping related papers. The close connection of the top twenty most influential authors, men and women, to the World Anti-Doping Agency in some professional capacity suggest that the Agency have an influence on the anti-doping research beyond directly providing funding. In terms of geographical regions, publications with female authors were dominated anti-doping research in Italy, Romania, and Spain. In research networks to date, women have outperformed male authors in information centrality, which means that women in anti-doping research have had higher level of control over the information flow in the field than their male counterparts. The results of this study confirm the potential of bibliometric approach in the identification of emerging research topics and quantifying gender differentiation in the field of anti-doping. Due to their higher information centrality, women are better positioned for problem-focused multidisciplinary research both within anti-doping community, and with researchers in cognate fields. Bibliometric analyses have proved to be a powerful tool for monitoring and advancing anti-doping research impact via identifying new avenues for multidisciplinary work, better gender representation, and diversity.

## Introduction

Analysis of place and role of women in sciences has considerable traditions (e.g., Thistlethwaite, 1959), and it became conventional wisdom, that “*Women scientists [have been] long underrepresented, underpromoted, and underpaid in their fields*” (Vetter 1976, p. 713). Progress have been made toward gender equality. Yet almost half a century later, a comprehensive report by a prominent publishing house still talks of gender inequality in research (Elsevier, 2021). Despite the encouraging signs of improvements, this study showed that women across countries and fields publish fewer papers, are less mobile, and less likely to be involved in international collaborations. On the other hand, the study also found that a slightly larger proportion of the research outputs by women are highly interdisciplinary than scholarly outputs of men. Health, life sciences, and social sciences are among those fields where women have the highest representation, which makes anti-doping research an intriguing field for exploring the presence and solutions for the “genderedness” of academic research, as well as unique “gendered” potentials.

In connection with sport policies and principles of good governance, studies focused on the importance of gender quotas on sport boards or the need for gender equity (e.g., Henry and Lee, 2004; Knoppers and McLachlan, 2018; Moura et al., 2020; Piggott, 2021). Even if gender may not be directly associated with principles of good governance (Parent and Hoye, 2018), gendered perspectives (inequality, standpoint, power) can have an impact on research priorities, agendas, approaches, and interpretations of findings, as well as funding allocations.

Specific to anti-doping research, two competing forces impact gender equality. On the one hand, the “gendered structure” of academic research and publishing tend to make women less visible, their voices less heard, and their career progression stunted (Lundine et al., 2018). On the other hand, anti-doping research—like most applied fields that center on complex issues and span across many subject areas encompassing sciences as well as social science disciplines—is thought to be highly interdisciplinary where inter- and multidisciplinary approaches are encouraged (Petroczi and Naughton, 2011; Viret, 2020). Therefore, in addition to being beneficial for advancing anti-doping, the high degree of inter- and multidisciplinarity of anti-doping research may also promote gender equality at a greater rate than in other fields. Parallel, global sport governing bodies (e.g., the World Anti-Doping Agency, International Olympic Committee, international sport federations) and sport organizations in Western, developed countries are committed to gender equality in terms of representation in committees, expert panels, and working groups (Pollack and Hafner-Burton, 2010; WADA, 2018). If the general gender-gap phenomena are valid for anti-doping related research, this means, that in this field there are considerable intellectual capacities, the better utilization is important to create more equal opportunities for the women scientists (Rosen, 2017), enhancing the spectrum of approaches (Otsubo, 2008), applied to study anti-doping problems, and integrating the possibly partly-utilized intellectual potential.

### Bibliometric Approach

Advances in computational social science and the development of different analytical methods—such as bibliometrics, scientometrics, and informetrics—can considerably enhance our knowledge on gender inequality in research, development, and academic publishing. Pioneering the field, Pritchard (1969) defined bibliometrics as “the application of mathematical and statistical methods to books and other media of communication” (1969, p. 348). Thirty years later, Glanzel observed that “bibliometrics is one of the rare truly interdisciplinary research fields to extend to almost all scientific fields” (2003, p. 5). This approach, however, is not to be conflated, or confused, with traditional literature reviews where the aim is to critically review, assess, synthesize, or pool research evidence together for a more robust observation of a phenomenon, and it is often driven by a specific research question about this phenomenon. In contrast, bibliometrics is a field of science, that focuses on quantitative aspects of measurement of scientific research output (Van Raan, 2019). As such, bibliometric methods facilitate the exploration of the conceptual-thematical structure, trends, and dynamism of the field of science by applying mathematical and statistical methods such as statistical-network theoretical modeling of the referencing, text-similarity, and authorial relations of the scientific literature.

Bibliometrics, scientometrics, and informetrics are closely related metric terms. With each of these terms featuring various definitions in the literature, these terms are used to describe similar and overlapping methodologies in science studies (Hood and Wilson, 2001). Bibliometric analyses rely on bibliometric indicators to a specific field of science, thus bibliometric studies apply mathematical and statistical methods to describe the different aspects of scientific communication. During the development of the field of bibliometrics, the main elements of bibliometric analysis have been defined as database compilation, consistency and accuracy of the data, data fields, search options, and analysis and use of metrics (Thompson and Walker, 2015). The application of contemporary bibliometric principles covers three sub-areas, namely methodology research, scientific disciplines, and science policy (Glanzel, 2003).

In the present study, we focus on the applications of bibliometric methods to anti-doping sciences, therefore it could be categorized under the sub-area of scientific disciplines. With bibliometric analyses, we can go beyond perceptions or anecdotal evidence, and formulate adequate methods for improvement, highlighting central points, “hot topics”, and best practices.

### Bibliometric Approach in Sport-Related Research

Although bibliometric analysis is not completely alien to sport science, it is still far from being fully utilized to advance the field. To date, bibliometric studies have been conducted in several fields of sport science such as factors influencing sport performance (e.g., Bilgiç and Işin, 2022). Its applications have been extended to sport management issues (e.g., Shilbury, 2011; Ciomaga, 2013; Belfiore et al., 2019; Baier-Fuentes et al., 2020), sport economics (Santos and García, 2011), innovation in sport (Ferreira et al., 2020), selected sports (e.g., Ibáñez et al., 2021; Millet et al., 2021), sport and exercise psychology (e.g., Lindahl et al., 2015; Clancy et al., 2017), physical activity and aging (Müller et al., 2016), and sport nutrition (Kiss et al., 2021). Specific to the anti-doping area, only a few bibliometric studies have been completed. The study by Agulló-Calatayud et al. (2008) identified key research centers and authors of scientific articles on anabolic steroids, whereas a working paper by Engelberg and Moston (n.d.) focuses on doping-related papers published in sport management journals.

### Women's Share in Academic Publication

Sex/gender disparities in academic productivity were studied based on the application of standard bibliometric indicators (e.g., publication productivity in terms of the number of publications and authors, citations, collaboration patterns, key author positions, and productivity by country) and their distributions (Halevi, 2019). A high number of bibliometric analyses have been conducted in recent years on gender disparities in different fields of sciences, such as women's contribution to science in life sciences (DesRoches et al., 2010), medicine (Pashkova et al., 2013; Henderson et al., 2014), economy (Maske et al., 2003), astronomy, immunology and oceanography (Leta and Lewison, 2003), psychology (D'Amico et al., 2011), and criminal justice and criminology (Snell et al., 2009). From the results and conclusions of academic research, an increasing body of evidence is drawing on women's participation in science. Regarding publication productivity, it is evident that women researchers publish less compared to male researchers, but citation patterns show a more complex picture. There are no differences in the citation patterns between genders in general, but a cross-disciplinary bibliometric study showed that papers with female authors in key positions (sole authorship, first- and last-authorship) are cited less than those with males in key positions (Larivière et al., 2013). The study of Dehdarirad et al. (2015) provides an overview of how research output in the field of women in science, in general, has developed from 1991 up until 2012. They acknowledge the outstanding role of women in education and educational research, psychology, information and library science, computer science, business and economics, and women's studies, but they underline a high degree of multidisciplinarity. The authors emphasize that besides bibliometric indicators, different factors (factors related to gender bias such as family-related issues, sociocultural factors) need to be incorporated when analyzing women's productivity and gender biases in science. Women's higher contribution in multidisciplinary research is also demonstrated by the Elsevier report (2021).

### Women in Sport Science Research

A handful of studies on sport sciences focusing on the representation of women in terms of authorship, membership in editorial boards, or academic positions is also available. Studies on gender trends in authorship characteristics in the field of sports science have mixed findings but with an overall trend toward a larger representation of women authors. For example, one study by Mujika and Taipale (2019) examined the gender differences in sport-science authorship. Based on a simple analysis of female and male authors in the first five issues of one sport science journals, the International Journal of Sports Physiology and Performance, published in 2019, they found that only 13% of the authors were women. The authors encourage sports scientists to take sociocultural biases into account during their academic activities (e.g., in selecting speakers for international conferences, reviewers for papers, choosing co-authors and collaborators).

The changing role of women in sport-related medical science is relatively well-investigated. Chang-Yeon et al. (2019) examined the relationship between gender and authorship in orthopedic sport medicine literature between 1972 and 2018 to show how the proportions of female authors in different authors' positions (first, second, middle, and senior authors) are evolved. Altogether, 16.6% of the authors in the sample were female. Whilst it is quite low, the analysis showed that there has been a significant increase in the proportion of female authorship (from 2.6 to 14.7%) in orthopedic sports medicine, the increase of almost 7-fold within the 46-year time frame. Publications with female authors were two-thirds the volume of male authors overall, however, female authors were more likely to be in middle authorship position.

Related to sport medicine, Loder et al. (2021) carried out a bibliometric analysis of the English musculoskeletal literature over the last 30 years. There were gender differences in the first and the corresponding author position, the percentage of female first authors increased from 10.8% in 1985–1987 to 23.7% in 2015–2016, while the percentage of female corresponding authors changed from 8.9% in 1985–1987 to 18.9% in 2015–2016. In addition, differences were shown in the first and corresponding author gender by journal type, specific journal, decade, and geographic region. Notably, female corresponding authors and female first authors were more common in the basic science group compared to the clinical group. The study of Dynako et al. (2020) analyzed the bibliometric and authorship trends in two representative American sports medicine journals, namely the American Journal of Sports Medicine (AJSM), and Arthroscopy in the last 30 years. The average percentage of female first authors was 13.3% for AJSM, increased from 4.7% in 1986 to 19.3% in 2016. For Arthroscopy, the average percentage of female first authors was 8.1%, increased from 2.8% in 1985/1986 to 15.7% in 2016. The AJSM had an overall greater percentage of female authors. To evaluate authorship trends, Ryan et al. (2020) examined articles published in Sports Health journal between 2009 and 2018 for the number of authors, and the presence of female authorship among others. The percentage of publications with at least one female author increased throughout the study period, from 52% in 2009 to 64% in 2018. The authors highlighted that the Sports Health journal continues to show high rates of female authorship compared with other various journals.

### Aims

Overall, the presence and performance of women in science in general and women in sport science, in particular, have increased significantly in recent decades, but gender-related differences still exist and remain a global phenomenon in the field. In the sport science literature, including the anti-doping sciences literature, gender footprint has not been widely investigated in terms of combining and linking the gender-related dimensions of sport science in a structured and systematic way encompassing multiple subject areas and subfields. To our best knowledge, there is no original article that specifically examines the role of women and provides a quantitative analysis aiming at gender differences in anti-doping sciences. With this study we aim to address this gap and—using state-of-the-art scientometric analyses—address the following research questions:

1. What is the contribution of women to anti-doping research?
2. What are the characteristic trends in women's contribution to anti-doping research over time?
3. What is the relational structure of gender aspects of country-related, authorial, topical, and methodological features?
4. What is the extent of recognition and impact of women in the related literature?
5. In what way are women “do better” than men in anti-doping research?

## Materials and Methods

Constructing the relevant literature corpus was challenging in many ways. First, identifying research outputs in and field delineation of the anti-doping research was one of the most important and critical steps, thus data collection and database construction formed the basis of the analysis. Anti-doping is a relatively new and emerging field that spans across multiple scientific fields, with a significant proportion of research being interdisciplinary. The second challenge involved the identification of male and female authors from bibliometric information.

### Data Collection

To delineate the anti-doping research field, bibliometrics-aided retrieval was used in line with the methodological paper of Gal et al. (2015). As a first step, a core dataset was created based on a core journal and search terms, then a broader dataset of publications/research outputs was compiled via the use of broad search terms, and finally, citation-based similarity measures between the documents in these datasets were applied in order to gain a final dataset with a high degree of precision.

For data collection and field delineation the Web of Science Core Collection databases were used (including the Science Citation Index, the Social Science Citation Index, and the Arts&Humanities Citation Index). Although many databases are now available for bibliometric analysis (e.g., WoS, Scopus, PubMed, etc.), a standard choice in bibliometric studies involves the WoS dataset as the Gold Standard. Irrespective of coverage issues, which are deeply influenced by indexing policies for these proprietary databases, WoS is usually considered to fulfill the relevant set of criteria for mapping purposes: (1) High-quality standards in indexing, representative coverage, (2) multidisciplinary, and (3) rich characterization of bibliographic records.

The basic documents of the data collection (core dataset) were obtained on one part from the WoS database with a search query of “anti-doping^*^ OR antidoping^*^” OR “anti-doping”^*^. The search terms were applied to the title only in WoS. One the other part data were obtained from Acute Topics In Anti-Doping book as one of the core literature. No limitations were placed on the dates of the searches, the final data of data collection has been 20. October 2021. The search resulted in a core set of 572 publications. To extend the information content of the core set, we created a broad search term using the following query: “doping control^*^” OR “doping prevention^*^” OR “doping-free sport”^*^ OR “clean sport^*^” OR “drug-free sport^*^” OR “anti doping in sport^*^” OR “fair sport^*^” OR “WADA^*^” OR “fair play^*^” OR “anti-doping^*^” OR “antidoping^*^” OR “anti doping^*^” in (Topic) in WoS database. To identify relevant anti-doping-specific search terms sport science journals, systematic reviews, highly cited research papers, and WADA documents were reviewed. The broad search term set included 2390 research papers. To increase precision, one of the authors who is an expert reviewed the title of publications of a random sample of the broad search term dataset. Publications were excluded from the core and broad search term dataset when the expert excluded the publication or the publication was written related to animals.

**Figure 1:**
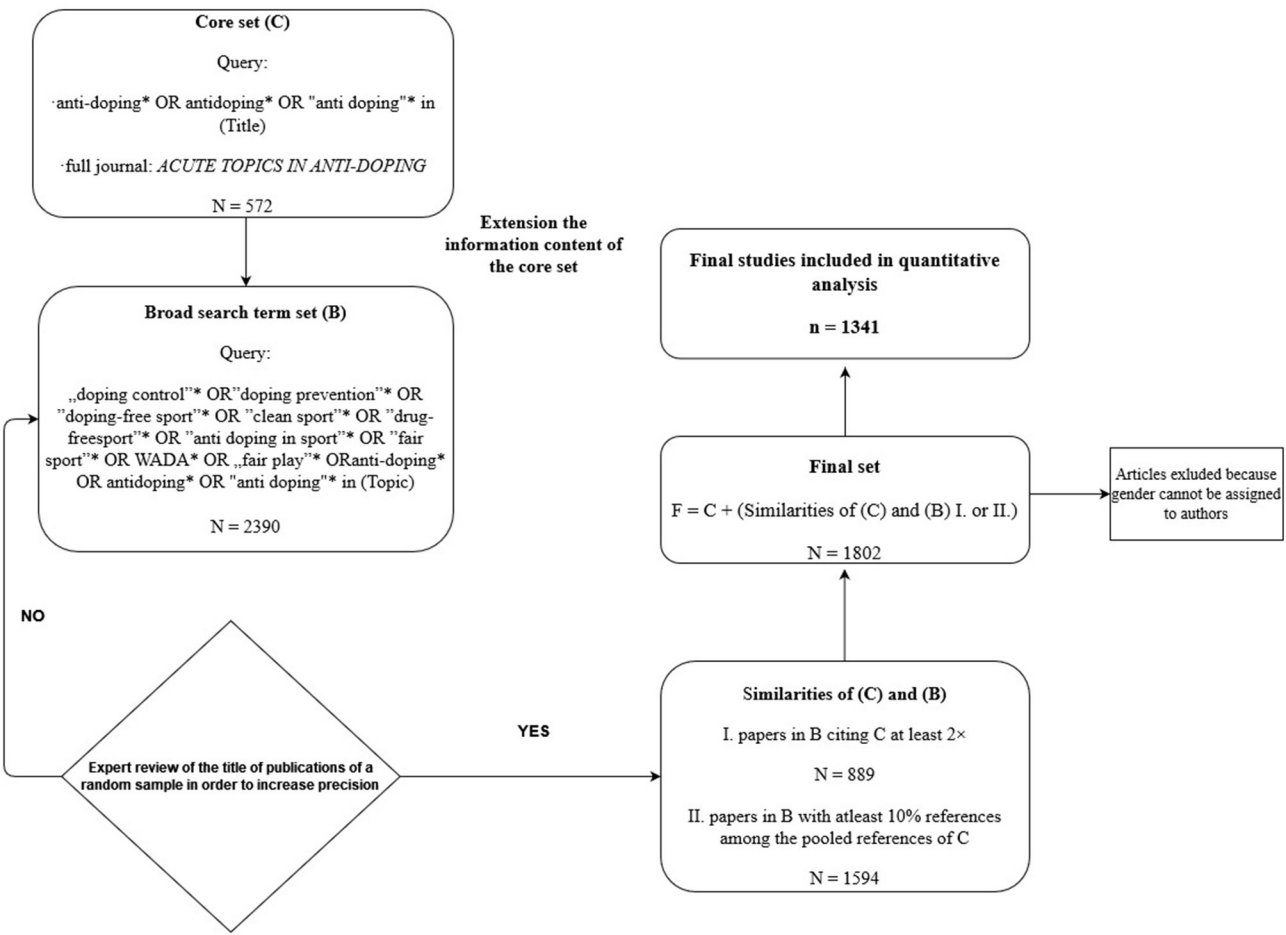
Flowchart of database construction.

Then citation-based similarity measures were applied in the core dataset and the broad search term set, the review of random samples led to the selection of publications of two combined datasets: group I. included papers in the broad search term set citing the core dataset at least twice (*N* = 889). To group II. dataset belonged papers in a broad search term set with at least 10% references among the pooled references of the core dataset (*N* = 1,594). Publications from the core dataset and similarities of the core dataset and the broad search term set (group I. or II.) were included in the final set, thus ensuring that only the relevant topics are included. After eliminating duplications, the final set included 1,802 publications, with dates of publication ranging between 1998 and 2021. After excluding studies to which genders cannot be assigned to authors 1,341 studies were included in the analyses. Figure 1 illustrates the identification workflow of relevant research papers.

The database of funded research projects was constructed from the publicly available records of successful grant applications (https://www.wada-ama.org/en/funded-scientific-research-projects and https://www.wada-ama.org/en/social-science-research-projects-0). At the time of the database construction, applicant details were not available thus we had to limit the researchers to the principal investigator. Because more details are now available, we will address this limitation in future studies.

### Sex/Gender Identification

As a first step toward gender identification, names of authors were cleaned: abbreviations and symbols were removed and abbreviated first names were rewritten in full (first name and last name) form. Middle names were also included in full names. Full author names were retrieved from the bibliographic data downloaded from the WoS databases, which contains a dedicated field for representing the complete name of contributing authors, and used to classify authors into binary categories as male or female. To assign genders we used Gender API (available from https://gender-api.com/en/), which is one of the biggest platforms on the internet to determine gender by a first name or a full name, their database contains 6,084,389 validated names from 191 different countries. Alongside the full name of the authors, we also identified the country assigned to each author from the publications because specifying the queries by adding country code is increase the accuracy of the result. Gender API (value is ranging between 0 and 100) provides an accuracy value in the query result. The final dataset contained 3,628 author names, from which we were able to identify 2,415 full author names. Of these, 2,346 (97%) authors' gender was assigned with an average accuracy value of 93.83. In the case of the missing values, the first name and middle name were abbreviated, and we were not able to resolve these abbreviations. The assigned gender category of the top 70 authors were confirmed by one of the authors who is a senior anti-doping researcher.

We are aware of the high theoretical importance of differentiation between categories of sex and gender (Johnson and Repta, 2012; Tannenbaum et al., 2019). Our studies have been based on sex differentiation of scientists, informed by their first names, that is why our study is sex rather than gender-based. At the same time, we analyze a gender-related problem, using gender-differentiation as a social construct (Kaliyath, 2016), that is why in the current paper the “sex” and “gender” words are used interchangeably.

### Data Analysis

**Figure 2:**
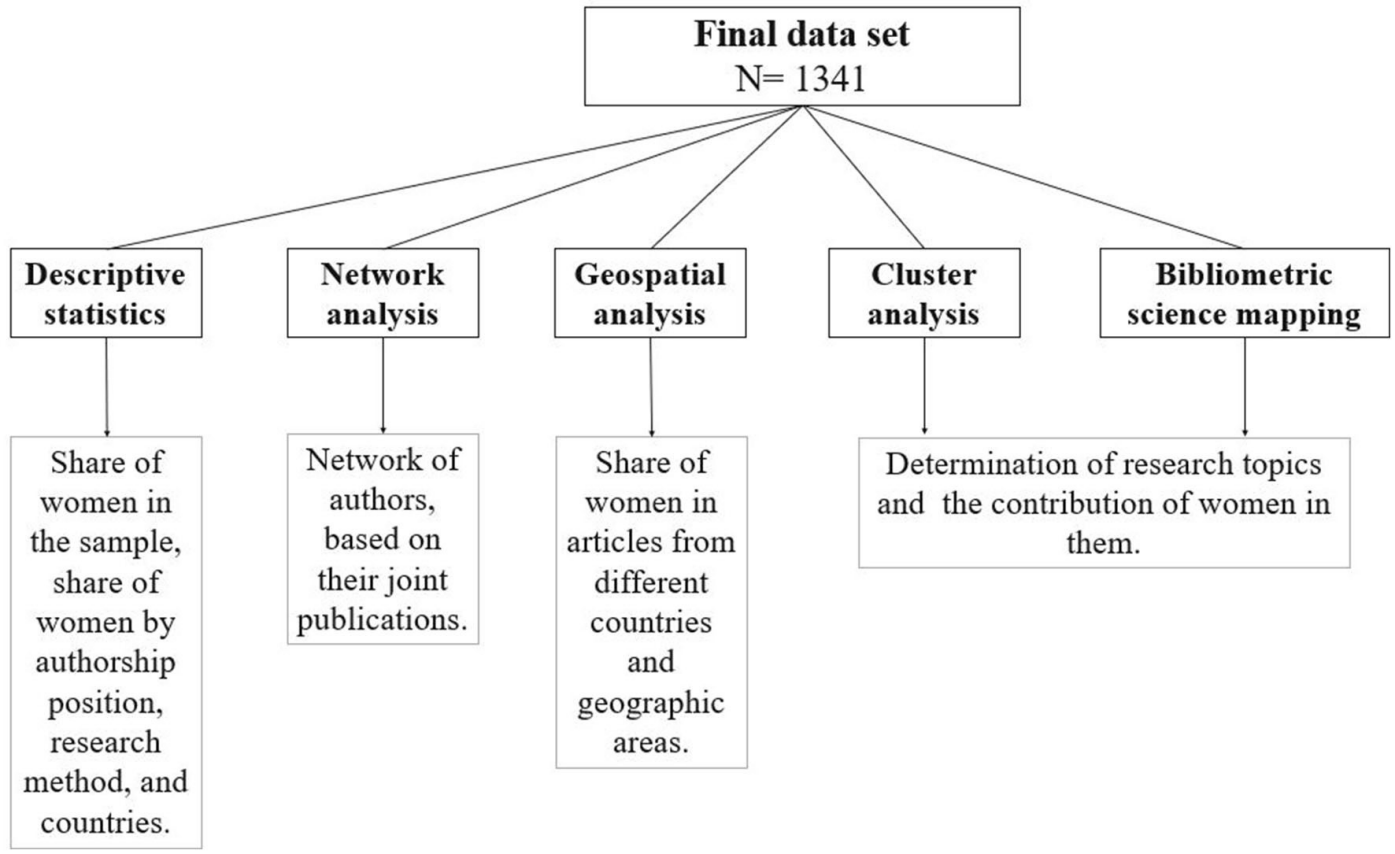
Workflow of the analytical framework.

Bibliometric analysis of documents, registered on WoS database, classification of different publications on the basis of topics and country, funding information, determination of the role of women in different papers. We have applied a triangulation approach. The use of different methods offered the benefit of analyzing the problems from different angles. Data analysis included geospatial analysis to reveal the contribution of women by geographical areas, cluster analysis to uncover emerging topics as well as the number and authorship position of women in them, and network analysis to create the authorship network and examine the presence and role of women in the network (Figure 2).

Topic identification, -or in this case, the clustering of publications into thematic groups- was based on the classical and validated bibliometric method of bibliographic coupling (BC). BC is designed to estimate the cognitive proximity between documents based on the relative amount of their shared references. The main idea behind this approach is that the more references two documents share, the more akin these documents are regarding their thematic focus. The similarity measures of BC usually uncover well-recognizable, specific topics inherent in the discourse. Our procedure consisted of the following steps:

1. Measurement of thematic document similarity. In the first step, we obtained the similarity matrix of documents based on the Jaccard similarity of their reference vectors.
2. Creating a document similarity graph. Upon the matrix, a document similarity graph was generated, with weighted edges representing proximity between them.
3. Graph-based clustering. In the last step, the document graph was subjected to a community detection algorithm (Louvain algorithm), based on modularity optimization. This yielded the densely connected communities (clusters) of papers, that could be deemed the emerging topical clusters of the discourse.

In data analysis, we followed the general workflow of bibliometric studies (Guler et al., 2016), including the calculation the Lotka's Law, which describes the frequency of publication by authors in a given field of science (Lotka, 1926). Bibliometric analysis was carried out by R statistical program (R Core Team, 2020). Cluster analysis as the development of the research field over time has been analyzed based on an algorithm, developed by Van Eck and Waltman (2010), and operationalized in CitNetExploere software.

## Results

### Anti-Doping Research Within Sport Sciences

**Figure 3:**
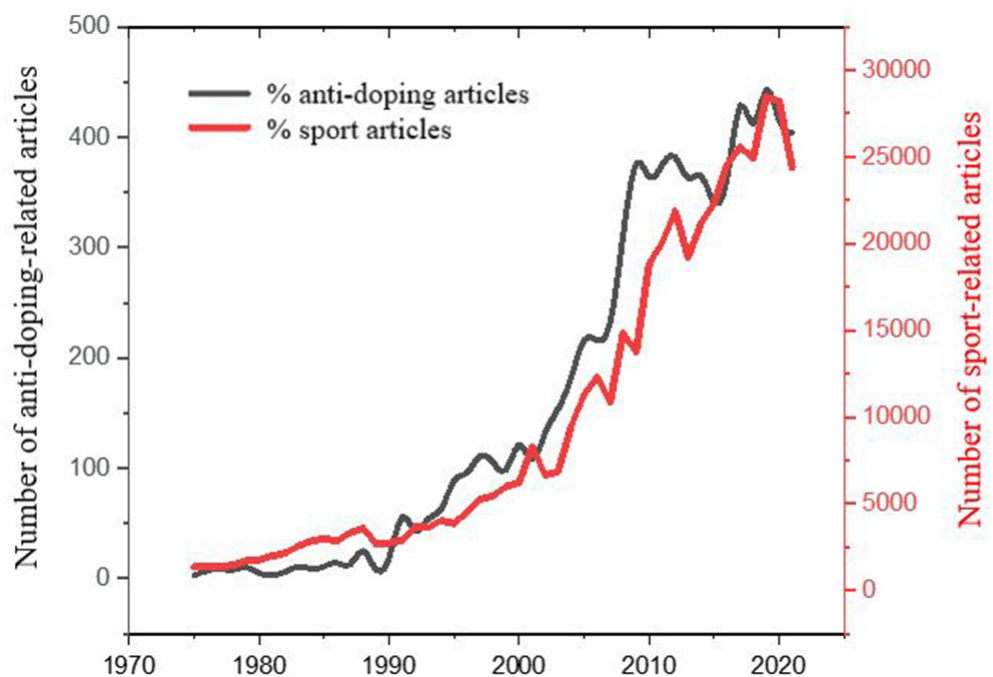
Trends in research outputs between 1975 and 2021 in sport and anti-doping studies.

Because the current study focuses on the role of women in anti-doping research, it is important to consider the general context and the position of anti-doping-related research in sport science. To analyze the trends of the share of these publications and the absolute number of them, we have downloaded the number of bibliographic units, registered in Web of Science (WoS) one of the worlds' most acknowledged academic databases (Martín-Martín et al., 2018; Liu, 2021) as units, attached to the field of science “Sport”. The results are depicted in Figure 3 and Supplementary Figure 1. There is an exponential trend in increasing sport related publications between 1975 and 2021. The number of anti-doping publications had also been increasing, however the share of anti-doping articles from all sport-related articles has been three times higher in the last decade.

### Basis Statistical Features of the Database

The original dataset consisted of 1,802 documents. More than two-thirds of publications have been research articles (1,268), with 205 reviews. Due to the extremely rapid development of this sphere of sciences, we have taken into consideration the proceedings papers (*n* = 66). The inter-professional discussions are well-reflected in editorial materials, which is why we also included 76 of these. In total, 3,628 authors were included in the basic corpus. Their names appeared 7,445 times in the publications, with only 219 outputs (18% of the total number of documents) being single-authored. On average, there were 4.13 co-authors of one document. This relatively high number indicates the highly complex nature of the field.

With language limitations acknowledged, the scientific analysis of the anti-doping field appears to be highly concentrated in the so-called WEIRD (western, educated, industrialized, rich, and democratic; Henrich et al., 2010) countries in the Northern Hemisphere. Based on the corresponding author's countries more than four-fifths of the articles were prepared in Europe, the USA, Japan, and Canada. The share of documents with European co-authors was nearly two-thirds. Contrary to another field of science share of Chinese (2.4%), Brazilian (2.2%), Russian (1.3%) and Indian (0.6%) authors were exceptionally low. This fact can be explained by differences in anti-doping research and regulation in these states, mainly in Russia (Rutland and Kazantsev, 2016; Altukhov and Nauright, 2018; Ohl et al., 2021 and China (Hong, 2006; Yang and Leung, 2008; Lu, 2012; Tan et al., 2020).

**Figure 4:**
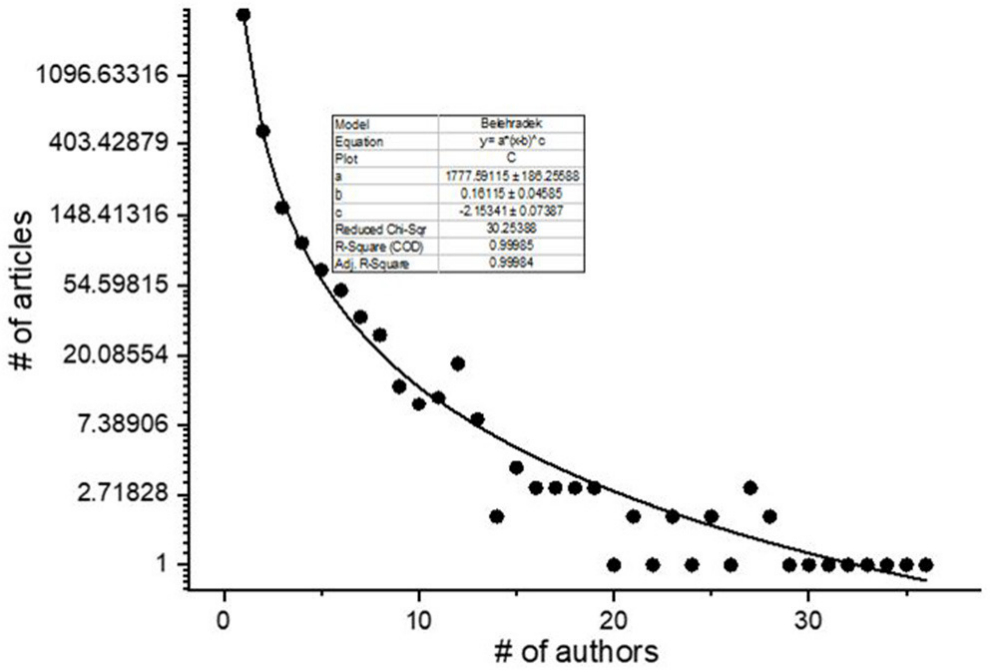
Distribution of all authors, by number of articles.

The distribution of actors shows a high level of concentration. The overwhelming majority of authors (2,595) appeared just one time in the corpus, no more than 70 authors produced more than ten articles. Fitting the Lotka distribution model generally applied for analysis of authors' distribution, the *r* square value was 0.85, the *c* value was 1.77 (Figure 4). This result highlights the fact that the anti-doping research community is a rather closed one.

### Contribution of Women to Anti-Doping Research

**Figure 5:**
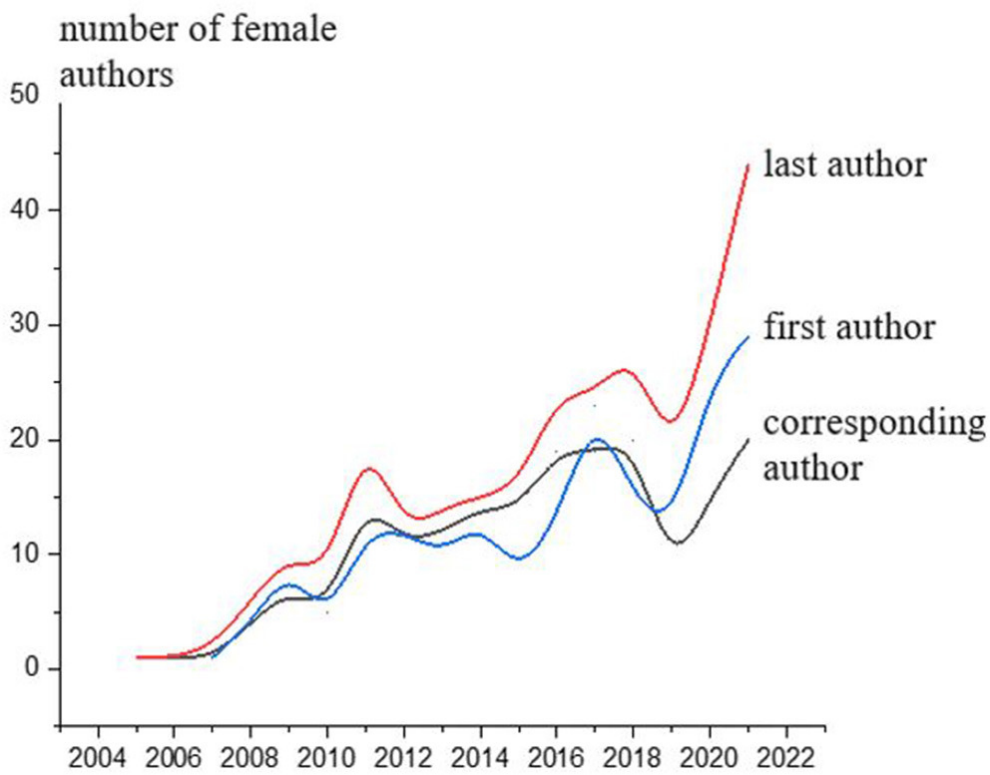
Number of female authors in key positions by year of publication.

Altogether the number of male authors was twice as high as the number of female authors with 1,100 being identified as female and 2,160 being identified as male in the data. The most important characteristic features of time dynamics of the absolute and relative position of authors are summarized in Figure 5. Over time, there is a noticeable increase in the share of female authors appearing in key positions (i.e., first or corresponding) among the authors. The number of female first authors increased from 1 in 2005 to 29 in 2021, while the number of female corresponding authors changed from 1 in 2005 to 19 in 2021 in the anti-doping literature. Parallel with the number of anti-doping papers, the number of female authors has changed significantly over time, from 2 female authors in 2005 to 182 female authors in 2021.

Analyzing the authorship position, large differences in the corresponding author position were observed. Publications with male corresponding authors were noted in 774 out of 991 papers. There was an overall greater share of male corresponding and other author positions in anti-doping-related studies. The share of women in key author positions was low. The average percentage of female first authors was 23.5%, and only 14.5% as last author, although the significance of the last author position (as senior project lead) is not universally used across different fields. The average author position was 3.45 in the case of female authors and 3.65 in the case of male authors (Table 1), which is partially influenced by the field (i.e., social science publications tend to have fewer number of authors than in sciences).

**Table 1:** Authorship position in the anti-doping related studies by gender.

**Gender**	**Other author (# papers)**	**Corresponding author (# papers)**	Average position (rank in the series of authors of a paper)^*^	Average weight (weight is calculated as an index: paper/author)^**^	**Corresponding authorship (share of papers)**
Female	473	217	3.45	0.22	0.19
Male	767	774	3.65	0.27	0.27

* *Average position: the mean value of author's placement in their papers' co-author list*.

** *Average weight: the mean value of author's share in their papers' co-author list (average of the inverse of the number of authors per paper)*.

**Figure 6:**
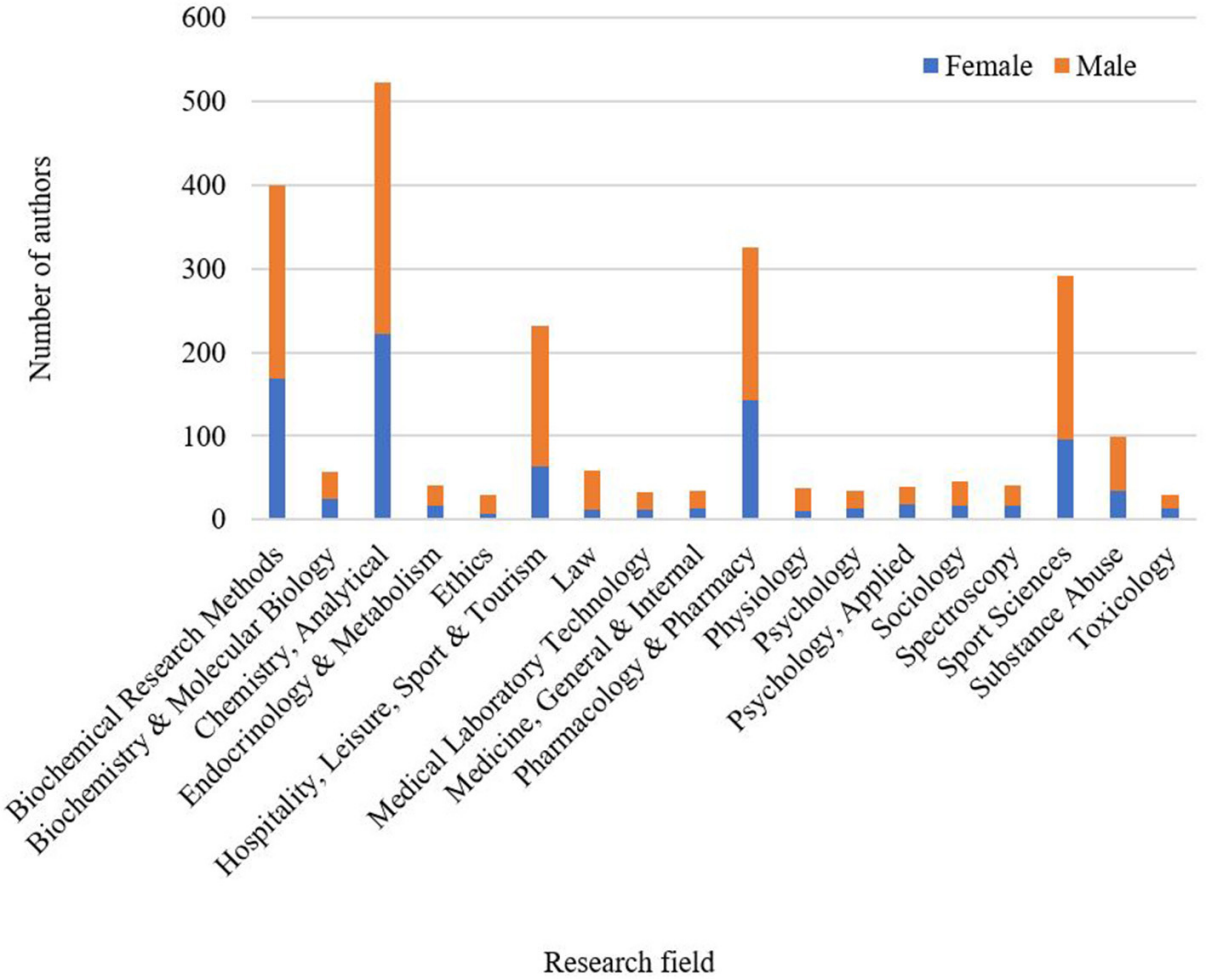
The proportion of female and male authors by research field.

Regarding the research field, most of the anti-doping-related paper was written in the field of analytical chemistry, followed by biochemical research methods, pharmacology & pharmacy, hospitality, leisure, sport & tourism, and sport sciences. The proportion of male authors was higher in all research fields, the number of male authors was twice as high in the research field of hospitality, leisure, sport & tourism (168 vs. 64), significantly higher in analytical chemistry (301 vs. 222) and sport sciences (196 vs. 96), suggesting that these research fields are dominated by male authors. The share of female and male authors was approximately equal in research fields that were present in a smaller size in the sample (e.g., applied psychology, nutrition & dietetics, toxicology). Figure 6 shows the share of women authors by research field.

**Figure 7:**
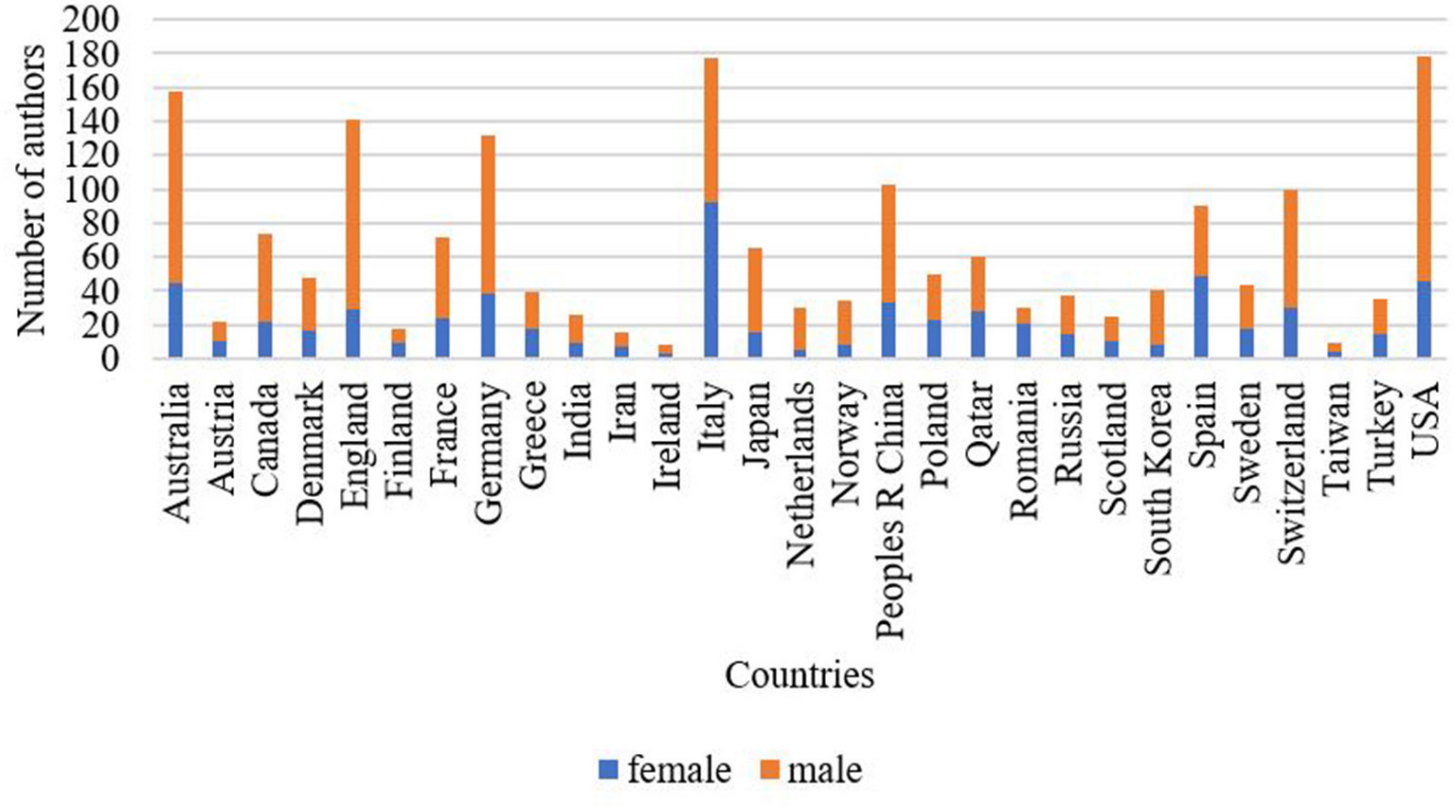
The proportion of female and male authors by country.

With reference to geographical distribution, the highest contribution to anti-doping-related research was from the USA and Italy, followed by Australia, England, and Germany. Specific to gender distribution within, the share of female authors was approximately equal to male authors in Austria, Finland, and Greece. The proportion of male authors was more than twice as high in the USA, Australia, Canada, England, Germany, Japan, and Switzerland. Publications with female authors were dominated in anti-doping-related research in Italy, Romania, and Spain, as the proportion of female authors was higher in these countries (Figure 7).

### Share and Performance of Women Among the Most Cited Actors

**Figure 8:**
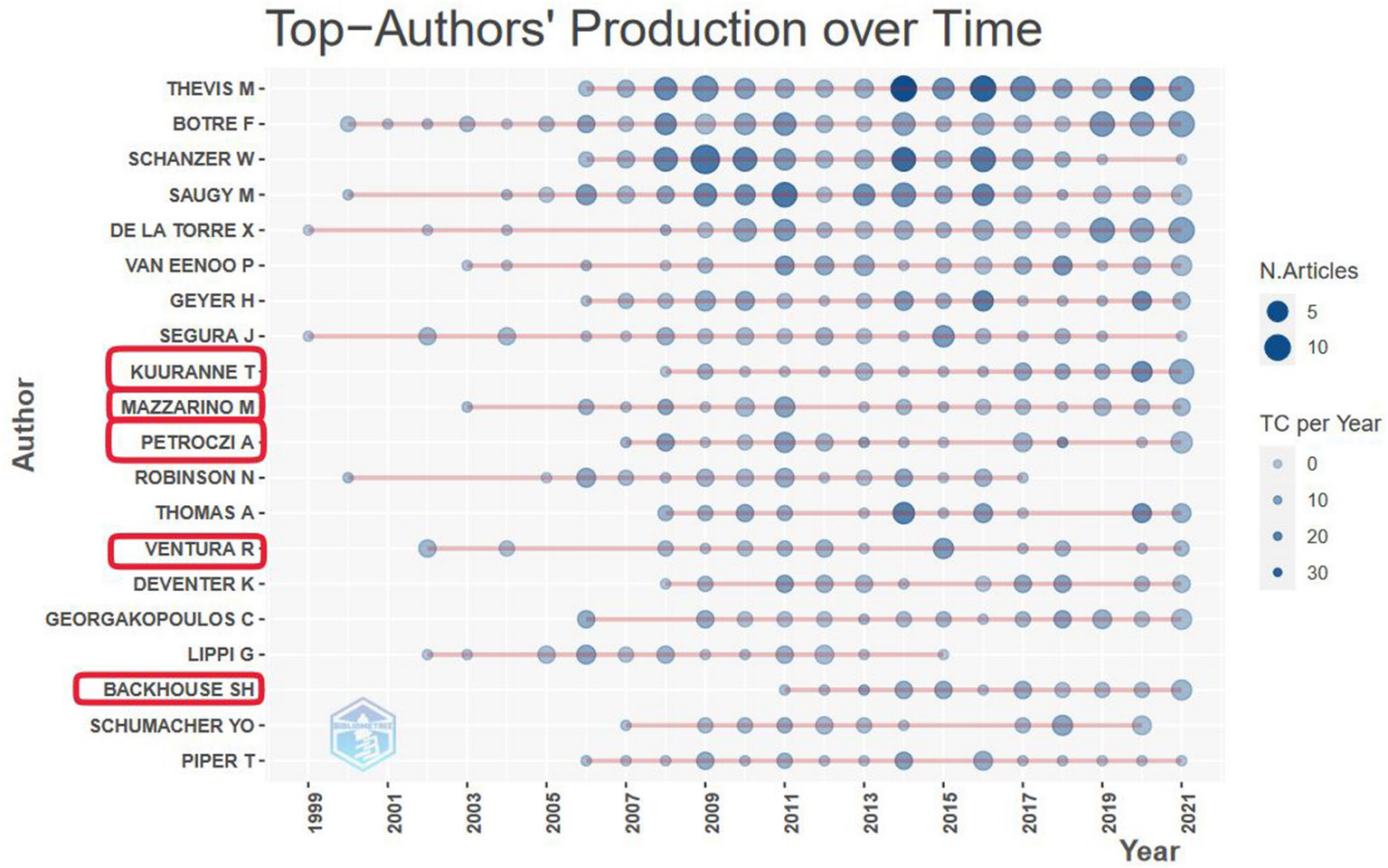
Yearly academic production (number of articles) and citation (total citation per year, TC) of the twenty most productive authors in the fields of anti-doping sciences. Women are indicated by a red circle.

Analyzing the structure of the most cited productive authors in the field, the results show that most of these researchers have more than one and half-decade research history and their stream of academic outputs is relatively stable. Among the twenty most productive authors, there are five women, which is a robust standing, but also a good indication of gender imbalances in this field. Upon closer scrutiny, it appears that these five women show a long-time, stable performance in the anti-doping research field (Figure 8). Of them, three (Kuuranne, Ventura, Mazzarino) work in doping control and testing, whereas the remaining two (Backhouse, Petroczi) research aspects of doping prevention (primarily focusing on behavior, prevalence, integrity, and anti-doping education).

In terms of academic impact, there were 19,214 citation links between the included papers. However, the time span of cited documents embraced more than six decades with the first cited document being published in 1957. Indicating the degree of maturity of the field, the overwhelming majority of cited sources have been written in the last three decades. This fact highlights that the academic research of anti-doping is a relatively new field of science, basic pillars of which have been hammered down by the current and still active generation of researchers.

**Figure 9:**
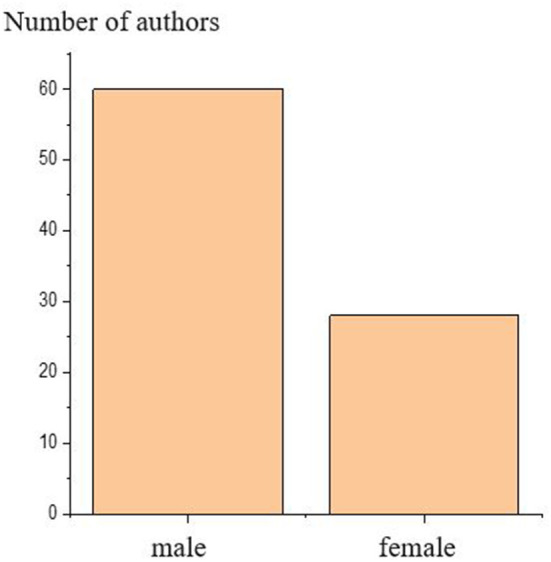
Number of authors by gender, whose name appears at least once among authors of papers, and obtained research funding from WADA.

To delineate the research impact further, we augmented the results from the bibliometric analysis with publicly available information on successful grant funding to the World Anti-doping Agency (WADA). Due to the limitation in the data, at this point, we made no attempt to make a direct connection between the source of funding and academic output(s) but we recognize the need for specialized research funding. WADA is one of the most important funding bodies for doping-related research, thus the distribution of the grants according to gender can be an important indicator of equilibrium or disequilibrium between the genders in case of allocation of financial resources. Striking differences in the number of female and male authors and male and female principal investigators for WADA-funded projects were found (Figure 9). Specifically, twice as many male researchers obtained funding for their anti-doping research than their female counterparts.

### Structure of Gender Aspects of Topical Features

Prominent research topics were determined via cluster analyses. Based on similarities of literature sources, six clusters were identified. The biggest cluster contains 771 publications and examines the social aspects of anti-doping research, followed by clusters focusing on the detection of prohibited substances and/or methods (doping), and the development of analytical methods. The most important characteristic features of the thematic structure are summarized in Table 2. Noteworthy is the nature of the largest cluster which essentially contains all social science outputs despite the fact that the cluster can be easily deconstructed by sub-fields such as sociology, psychology, ethnics, law, education, management, or governance. The formation of a single cluster of these fields suggests a high degree of permeability of the topic (doping and anti-doping) between the disciplines that concern about the behavioral aspect of doping.

**Table 2:** Characteristic features of clusters of publications, based on their sources.

**Cluster number**	**Size of the cluster (number of publications)**	**Description**
1	771	Social aspects of anti-doping and anti-doping policy formation
2	737	Development of chemical methods for detection and identification of doping
3	614	Detection of blood doping, the biochemical mechanism of doping
4	127	Pharmacology and pharmacokinetics in the detection of doping-agents
5	61	Anti-doping control practices, drug-testing methods
6	22	Role of pharmaceutics and pharmaceuticals in anti-doping; Application of cannabis, and cannabis-related products in sport

The structure of different clusters shows highly important aspects regarding the genders of the most cited actors. If we analyze a relatively small cluster (e.g., Cluster no. 5) it shows, that this cluster can be sub-divided into two components. One cluster (the larger one) deals with different methods of detection of doping and the smaller one deals with the issue of the establishment of an adequate regulatory framework. This cluster is a good example of the practical application of results, achieved in the field of doping identification and detection. It is a common feature of both sub-clusters, that the share of women is rather low among the most cited actors. However, in recent years some articles with women's participation have achieved considerably more attention in this cluster, than earlier publications by men (Supplementary Figure 2). Of course, this trend is likely to be driven by the focus of these research outputs and not the gender of the authors *per se*. What this perhaps suggests is the tendency of women researchers focusing more on emerging and/or “hot” issues; or the interdisciplinarity of the issues where women scientists generally do better than their male counterparts.

More analytical chemistry-oriented, large cluster (Supplementary Figure 3) features a considerable number of publications with women scientists. At the same time, no paper can be characterized by a preponderance of the women authors, and two papers achieved a 50-50 male-female ratio.

Theoretically, the position of the largest cluster, which deals with sociology and social psychology as well as the sport-policy aspect of anti-doping, is extremely important because this cluster comprises publications aiming to determine the key direction of anti-doping policy. In this case, a very high level of male-author dominance is noted with just a quarter of publications had been written with contributions from women. On a positive note, there is an upward trajectory showing that near the beginning of the second decade of the new century the relative frequency of the articles by or with female authors is increasing (Supplementary Figure 4).

### Women's Contribution to the Key Areas in Anti-Doping Research

Results of bibliometric mapping—another methodological approach to determine the key areas in anti-doping science—led us to further divide the publications in this database into 23 clusters, thus these results provided a more detailed view of the cognitive structure of the field. The absolute and relative size of the topics, derived from the number of publications belonging to a cluster, offers valuable insights into the research trends in anti-doping sciences, and the contribution of women within this field. The three most dominant clusters are: “Anti-doping education and doping prevention” with 172 publications, followed by a topic with 120 publications “Detection of blood doping/Athlete Biological Passport” and topic “Detection: Steroids/stimulants & narcotics” with 111 publications. The share of articles with at least one woman author was the highest in the cluster “Cannabis use as anti-doping rule violation” (0.96), the second-highest was the “Detection for cobalt and meldonium” (0.75). Women's contribution was higher in smaller clusters. In terms of change in percent point of the share of articles with at least one woman author clusters “Legal aspects of anti-doping,” “Implementation of the anti-doping regulations,” and “Doping control: new and special methods” have the highest positive change over time. The thematic structure and its important features by gender distribution are summarized in Table 3.

**Table 3:** The thematic structure of the field of anti-doping science and women's contribution within it.

**Cluster names**	**Total number of articles**	**Share of articles with at least one woman author**	**2012/2016 average of the share of articles with at least one woman author**	**2017/2021 average of the share of articles with at least one woman author**	**Change in percent point of the share of articles with at least one woman author**
Critique of anti-doping rules and implementation	26	0.06	0.12	0.00	−0.12
Anti-doping and ethics	80	0.19	0.15	0.33	0.19
Detection: hair analysis for steroids	5	0.25	0.00	1.00	1.00
Threats against integrity	95	0.30	0.31	0.23	−0.08
Detection of blood doping|Athlete biological passport	120	0.36	0.12	0.39	0.27
Detection: endogenous hormones	13	0.38	0.67	1.00	0.33
Detection: EPO/Blood doping	37	0.41	0.25	0.49	0.24
Doping control and treatment of asthma	40	0.41	0.48	0.26	−0.21
Legal aspects of anti-doping	15	0.43	0.17	0.54	0.38
Doping control and global harmonization	10	0.44	0.75	0.33	−0.42
Implementation of the anti-doping regulations	15	0.45	0.17	0.50	0.33
Doping control: new and special methods	10	0.50	0.00	0.33	0.33
Performance-enhancing substances in the society	29	0.50	0.67	0.42	−0.25
Role of pharmacists in anti-doping	12	0.52	0.67	0.58	−0.08
Anti-doping education and doping prevention	172	0.55	0.38	0.49	0.11
Advances in doping control: detection of endogeneous hormones (growth hormone, insulin)	41	0.60	0.62	0.46	−0.16
Gene doping	27	0.63	0.28	0.47	0.19
Detection: dried blood spot and alternative matrices	33	0.63	0.50	0.18	−0.32
Detection: Steroids/stimulanst & narcotics	111	0.65	0.58	0.50	−0.08
Dietary supplements and doping	41	0.66	0.42	0.46	0.05
Detection: endogeneous steroids and precursors (prohormones)	93	0.67	0.27	0.59	0.32
Detection for cobalt and meldonium	25	0.75	0.50	0.45	−0.05
Cannabis use as anti-doping rule violation	9	0.96	1.00	0.88	−0.13

### Structure of Gender Aspects of Authorial and Country-Related Features

**Figure 10:**
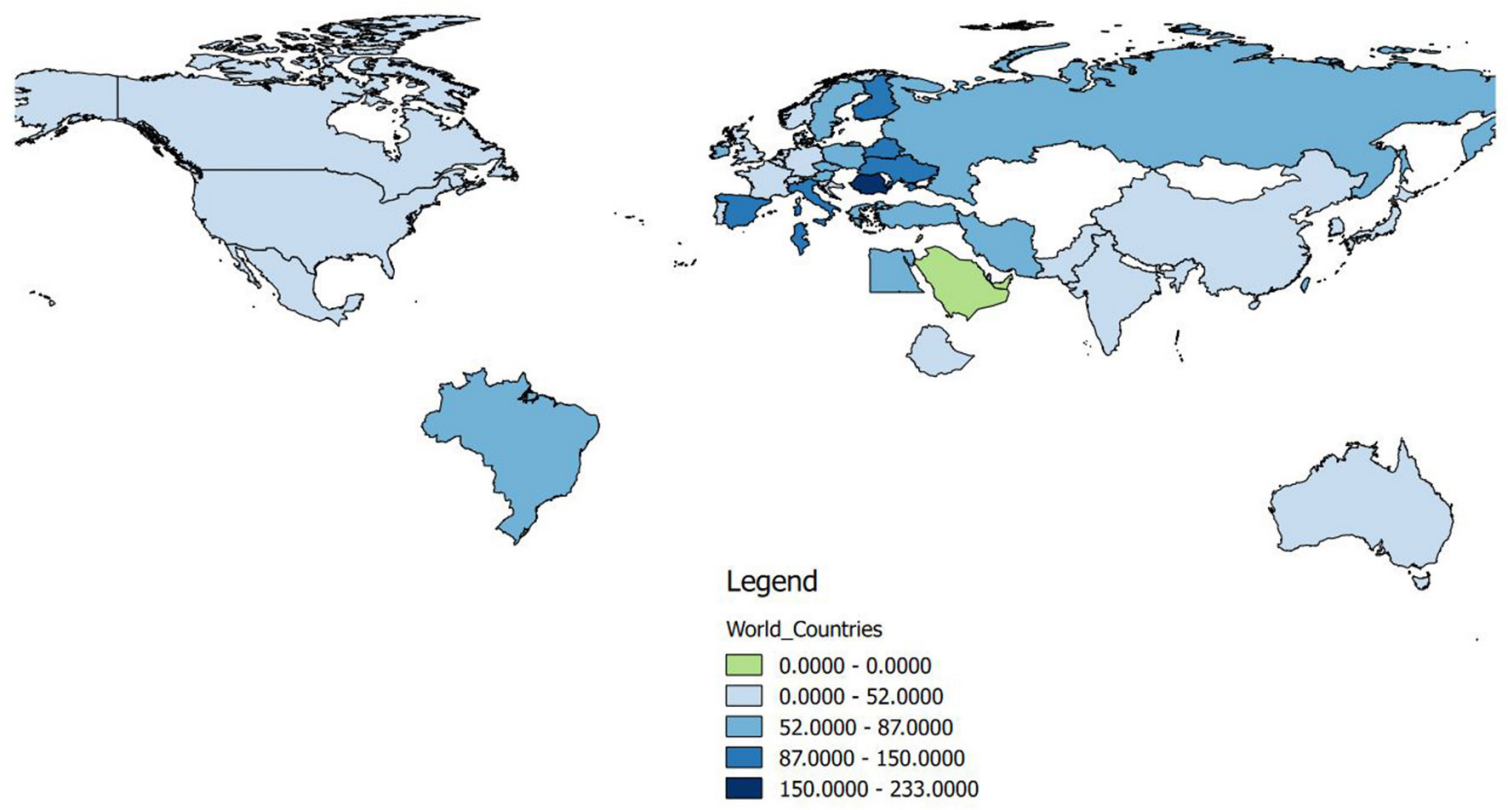
The average share of women authors in articles, prepared in different countries (in percent of male authors).

The share of women in articles from different countries and geographic areas shows considerable differences. Figure 10 depicts the relative values regarding the presence of women authors, the highest proportion of female researchers was in the Central-European region, and the lowest proportion was in the Middle East region.

### Authorship Network

**Figure 11:**
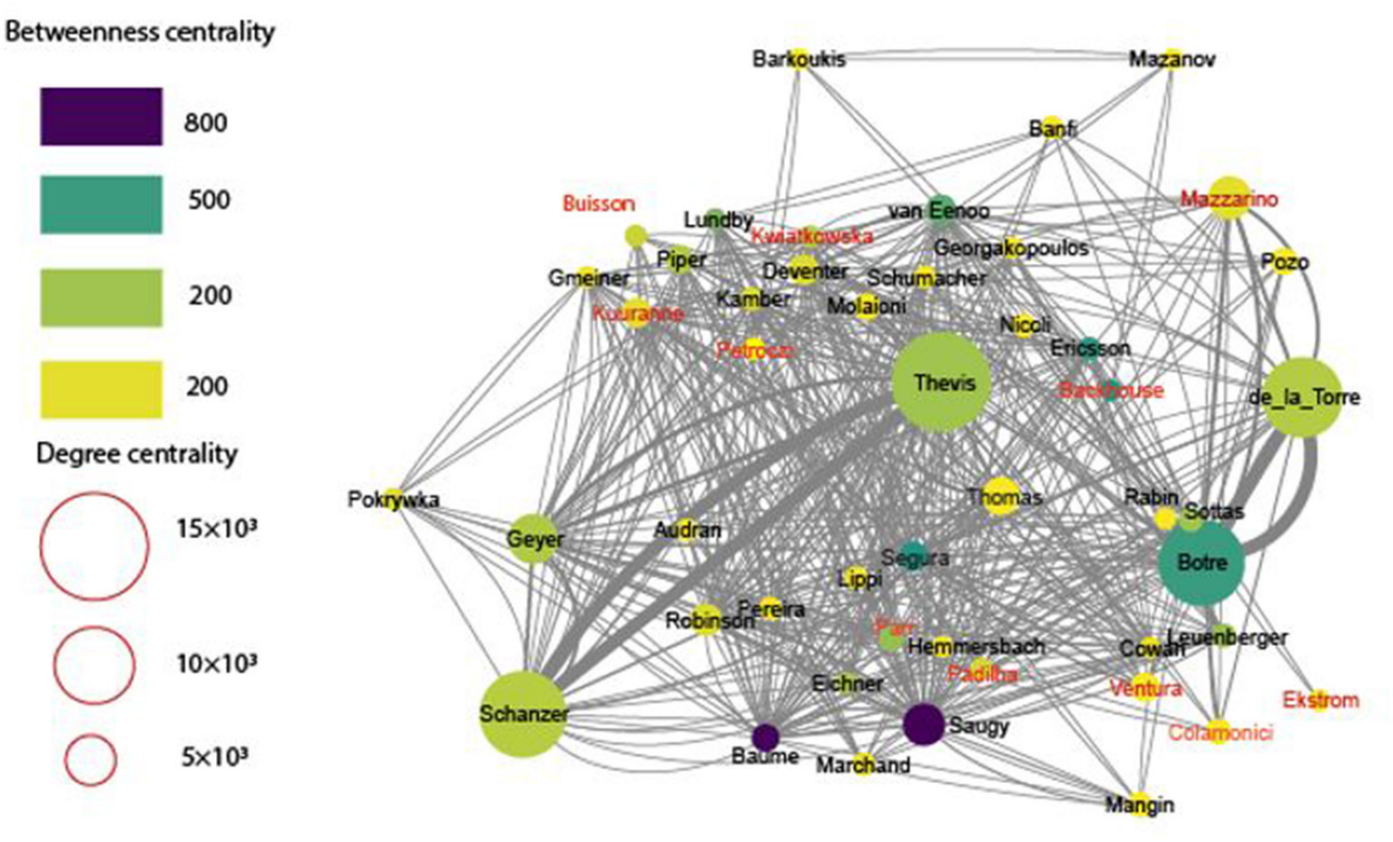
Network of key authors in anti-doping research, based on joint publications. The nodes (circles) indicate the authors, and edges (lines) mark the joint publications. The color of nodes is proportional with the betweenness centrality, the size with degree centrality, and the thickness of lines with the number of joint publications. The women authors are denoted in red.

To map research influence and academic impact via shared knowledge and expertise, we have analyzed and visualized the network of authors based on their co-authored publications. Based on the final dataset, we generated a network consisting of just 50 authors (Figure 11). This observation highlights the relatively low level of stability of cooperation networks between the key personalities of the research field.

Because there is no one indicator of the centrality of different nodes in a system (Tang et al., 2015), author networks are interrogated via a combination of different parameters. To determine various indicators of the centrality of authors as nodes, we obtained the following parameters: the betweenness centrality (Barthelemy, 2004), closeness centrality (Goldstein and Vitevitch, 2017), eigenvalue centrality, degree centrality (Pal et al., 2010), network centrality (Wang et al., 2011), information centrality (Rastogi, 2019) and local average connectivity based indicator. Each of these has shed new light on the centrality of the nodes, in our case of authors. The most important positions, determined by edge betweenness and degree centrality (indicators for characterization of the relative importance of nodes in the network, from point of view of the flow of connections among different parts of it), are occupied by men. Taken all together, only eleven women appeared as a node (individual) in the network, and only two of them (Backhouse and Petroczi) were from social sciences. Centrality measures for authors in anti-doping sciences are presented in Table 4.

**Table 4:** The indicators of the centrality of authors by gender (The scales are different, but the higher values indicate a more central role in the network).

**Indicators**	**Sex**	**Mean**	**Standard deviation**	**Independent samples *t*-test statistics, *p*-value**
Betweenness	Male	53.54	87.22	0.466, *p* = 0.466
	Female	44.07	24.35	
Closeness	Male	0.3761	0.09	0.141, *p* = 0.483
	Female	0.3925	0.042	
Eigenvalue	Male	0.04	0.05	0.899, *p* = 0.373
	Female	0.01	0.021	
Degree	Male	1810	3143	0.1006, *p* = 0.316
	Female	865	1221	
Network	Male	47.07	48.22	2.004, *p* = 0.051
	Female	16.76	13.44	
Information (homogeneity of variances hypothesis violated at *p* < 0.05 level of significance)^*^	Male	36.24	31.8	−2.767, *p* = 0.008
	Female	50.29	44.65	
Local average connectivity	Male	1656	155	0.302, *p* = 0.408
	Female	1462	1429	

* *Information centrality is a centrality measure of nodes (in this context), a variant of closeness centrality. It measures the relative drop in network efficiency (defined in terms of communication potential through shortest paths) caused by deactivating the node in question. Homogeneity refers to the assumption of the homogeneity of variance in testing the significance of the (mean) differences between women's and men's centrality values*.

Although the differences between male and female authors were visible in all indicators, due to the large standard deviations, statistically significant difference at *p* < 0.05 was not reached (Table 4), except for the information centrality indicator which shows a significantly higher value for women (*p* = 0.008). The significantly higher information centrality of women means that they have a higher level of control over information flow in the system.

## Discussion

The purpose of this study was to obtain an overview of the contribution of women to the scientific foundations of anti-doping, based on a rigorous, systematic analysis of international academic literature. To characterize the presence, role, and contribution of women to research outputs in anti-doping science over the last two and a half-decades, and to gain a comprehensive picture of gender trends in the field of anti-doping, we have applied a combination of different bibliometric approaches because they offer additional, complementary pieces of information, uncover hidden relations and perspectives to the gender footprint.

### Usefulness of Bibliometric Analyses

To reveal the scientific landscape of anti-doping and the positions of women within that landscape, we applied two different methodological approaches: cluster analysis and bibliometric mapping. The results confirm the significance of bibliometric mapping because the outcome of this method provides a more comprehensive view of the thematic structure of the field. On the other hand, the bibliometric mapping approach offers prospective ways of further research, identifying key actors within the research field, and contributing to an optimal allocation of intellectual and material resources of research.

Specific to anti-doping, this study showed that scientific output has been continuously rising in the last decades. Expanding the scope of study beyond women researchers and employing a different focus for either comprehensive or targeted enquiry, bibliometric analyses can offer a systematic map of anti-doping research activity and its impact to facilitate these focused and targeted reviews by creating a comprehensive overview, and handy clustered collection of the body of anti-doping knowledge and research output. The methodology is suitable to uncover hidden relations of anti-doping research that emerge from the scientific output, thus besides the revealing of thematic organization of the field, bibliometric analysis can complement experts' interpretation of the anti-doping research field as featured in traditional critical or systematic literature reviews.

Presented in a form of knowledge and impact (science) maps, bibliometric knowledge maps demonstrate the key elements of each research direction, the intellectual basis of them, and the conceptual structure of the scientific discourse. Therefore, bibliometric studies have multiple potential uses in anti-doping. Such studies can help organizations and funding bodies to minimize duplication of research, provide context for funding allocations, offer insight into how to improve their research programs, and inform decision-makers on the success of funding allocation strategies. Bibliometric analyses can identify key researchers and research hubs to facilitate collaboration and the dissemination and implementation of knowledge, opportunities for new collaborations for multidisciplinary work, and cognate subject areas that can bring fresh approaches, new expertise, and new methodologies to anti-doping. It can also detect the so-called “invisible colleges” (Newman, 2004; Palacios-Núñez et al., 2018) that may help or hinder research development.

### The Role of Women Authors in Advancing Anti-Doping

Like other gender studies in sport science, the presence and role of women in anti-doping science were under-represented. The number of female authors was half of the number of male authors in our analysis. As a whole, our results do not confirm the pattern observed elsewhere for health, life, and social sciences, which shows that multidisciplinary research areas have higher or the highest representation of women (Dehdarirad et al., 2015; Elsevier, 2021). However, in the social science subset alone, which appears to be highly interdisciplinary within social sciences, women researchers have been better represented. Generally, there were very few research outputs in anti-doping that crossed science and social sciences within a single study.

Furthermore, female authors were more likely to be in authorship positions other than first or corresponding. There were also differences in the citation patterns of male and female scholars, share and performance of women among the most cited actors were low. Our results are in line with the study of Larivière et al. (2013) who examined global gender disparities in science and showed that papers with female authors in key positions are cited less compared to publications with male authors in key positions. However, it is important to mention that citation differences are not directly because of the gender of authors but also the research field they are linked to. Females are involved more in “applied” and “practical” research which means that their impact is outside academia and could not be detected by citations. The number of citations is just one but not the only indicator of women's contribution to anti-doping research.

Regarding the authorship network, the most important network positions were occupied by men. This fact highlights that with some exceptions women have not been able to set up a considerable network in this men-dominated realm. Dehdarirad et al. (2015) showed that women had the highest contribution to the field of education and psychology among others. Education and psychology studies related to anti-doping research were important and dominant in more clusters such as in cluster “Anti-doping education and doping prevention” but in contrast to Dehdarirad et al. (2015), the share of articles with at least one woman author was relatively low at just 55%.

The presence of women in the authorship network showed great differences, especially in network indicators. Among the centrality indicators, the Information centrality indicator shows a significantly higher value in the case of women. This fact can be evaluated as a favorable one because according to Shan et al. (2018), actors in a network with higher information centrality have a higher level of control over information flow in the system. In our opinion, this relatively favorable position of women authors in the network can be explained by the fact, that women could be key actors in knowledge integration in anti-doping sciences.

Although women are under-represented in anti-doping, their share in scientific communication is increasing, the number of female authors is doubled in key positions within the 24-year time frame. The increasing share of women in the anti-doping field is especially important for three reasons: (1) this should be a natural contribution to the enhancement of gender equality, which is a natural part of the development of the societies and sciences. (2) The enhancement of women in science means an increase of intellectual capacities. This is especially important in an era when there is a new, and ever-increasing development of different methods of doping, threatening its integrity. (3) The integration of the feminist approach into the anti-doping realm can further enhance the anti-doping knowledge, research, and discussion by critical evaluation and reconstruction of the subject. Following the logic and argumentation of Israel and Sachs (2013) on the role of feminist science studies on climate change, in the case of anti-doping science feminists' approach (a) can contribute to enhancing the anti-doping activism, which is not built just on control and regulation, but is based upon the voluntary activity and self-conscience of athletes; (b) increased integration of standpoints and opinion as well as specific problems of oppressed, marginalized group of athletes (e.g., children, athletes from the third world), (c) contribution to the better understanding of doping-problem by the deconstruction of it and stepping beyond the traditional approaches, based on “wet” and “hard” science and ever-increasing control.

### Implications of the Observed “Gendered” Research Trends

The most prolific female authors appear to be successful in obtaining funding for anti-doping research. Having funding not only facilitates research activities but often provides support for research assistants and builds collaboration networks—both of which can have a positive impact on the number of publications. Specific to social sciences, international collaboration can generate more impactful research with larger and more comprehensive samples. Academic institutions that use bibliometric data to inform decisions about internal funding (e.g., for PhD studentships, seed grants, open access publication) and/or promotion should be aware of the gender disparity, which is exemplified in this paper but not limited to anti-doping science (Gender Equality in Research Innovation, 2021). Equally, countries, where governments are using some form of research evaluation to allocate research funding to academic institutions (e.g., the Research Excellence Framework (REF) in the UK, or similar central assessments in Australia or Italy), are also impacted. For example, in the UK's REF assessment, gender equality is monitored, but because the Unit of Assessments for REF submission is an institutional choice, gender equality indices are not comparable across institutions (Gender Inequality Is Still Baked in to the REF, 2020).

The under-representation of women in anti-doping research and their low share of research funding lock many women researchers into a vicious circle. Lundine et al. (2018) argue that “the gendered structure of academic publishing is both a reflection and a cause of women's under-representation and disadvantage in other areas of the scientific enterprise” (p. 1755). By receiving less research funding, women were not only handicapped in the race for high-impact research, but they became less visible in academic publishing, which then leads to being less likely to be invited as peer reviewers or editors for scientific journals, which then reduces their chances to obtain research funding. Results from the current study on anti-doping appear to confirm this pattern. Women researchers featuring among the top twenty authors have multiple research fundings from WADA and other sources, under their belts. Furthermore, most authors on this top-twenty list work or worked in WADA accredited anti-doping laboratories, which have a mandatory allocation of 7% or more of their operational annual budget on research (WADA International Standards for Laboratories, 2021, Articles 4.3.2 and 4.4.2.6), or linked to WADA in some other (academic) capacity such as serving on expert panels, working groups or filling *ad hoc* advisory roles for bespoke projects.

## Limitations

The current study had been focusing on the role of women researchers in anti-doping. The set of publications, used for the analysis was just a relatively small, but carefully curated part of the total publications dealing with the doping problem. Based on a larger sample, more exact mathematical models could be constructed to determine the most important influencing factors of place and role of women in anti-doping-related articles. If and when the number of publications increases, the application of more sophisticated methods of identification of the sex of authors could enhance the explanatory power of the models. Of course, we have tacitly supposed that the participation of women in academic articles is proportional to their role in research. The reality of this statement should be validated by more sophisticated, qualitative methods.

Overlaying grant data to key authors was purposefully limited to WADA-funded research. Although it is a limitation on the available information, we felt that relatively new funding avenues with a preference for science vs. social science (e.g., Partnership for Clean Competition, EU ERASMUS+ Collaborative Partnership grants) and *ad hoc* opportunities (e.g., research funding provided by the International Olympic Committee for 3 years) would skew the results. It must also be acknowledged that national-level funding for anti-doping is also available, but both the access to these and the level of funding available vary widely between countries and regions. Future investigation with a more nuanced analysis of the relationship between grant funding and academic outputs will contribute to research impact assessment, and thus be recommended.

Last, but not least we must stress that we have identified the gender of the authors on the base of their names. It is another question how accurately this reflects the authors' gender identity. Notwithstanding these shortcomings of the current study, we hope, that our research sheds light on an existing problem, and it will motivate other research activities for a better understanding of the participation and role of women in anti-doping sciences.

### Recommendations

We have seen that there is a considerable gap in the participation of women and men in anti-doping sciences. This situation is similar to other fields of sciences, hence (re)citing the general guidelines (e.g., National Research Council, 1991), strategies, and best practices (Coe et al., 2019) to improve gender inequality offers a rather limited added value. Instead, the field should utilize and build on the fact that anti-doping is a relatively new subject field, with pioneering researchers still active and most likely available for mentoring the next generation of researchers. Instead of solely focusing on compliance by artificially “ensuring gender balance” in recruitment, boards and expert panels, the field should focus on a long-term solution via the next generation of researchers, and beyond.

To facilitate this progress, two specific aspects of the anti-doping field should be taken into consideration. Firstly, the public and implicitly the political level of interest shows a large and relatively cyclical fluctuation. The important sport events (e.g., the Olympic Games) and some notorious doping scandals, like the case of Armstrong (Zurloni et al., 2015) increase the level of interest in the anti-doping topic. Under these conditions, it is a real danger, that the political decision-makers will be reluctant to allocate additional monetary resources for research and development. On the other hand, there is a rapid increase of challenges that need academically well-founded, non-partisan answers in this field. The continuous finance of these activities is a necessary precondition of efficient anti-doping regulation. Another relatively specific aspect of anti-doping research, as an academic field is a relatively low degree of overlapping between the “wet” sciences (analytical chemistry, pharmaceutics) and the research of social aspects of the topic. Consequently, the organization of interdisciplinary teams should have been a priority, while taking gender disparity into account. In fact, the findings of this research indicated that women researchers—through their better connectivity—are well-positioned to lead this line of research.

Secondly, by applying the model of the triple helix (Leydesdorff and Etzkowitz, 1998) to anti-doping research outputs, one can determine the set of theoretical possibilities (indicated by ABC triangle) which as—due to the socio-ethical, legal, or scientific conditions—limited a relatively small part (indicated by X, Y, Z triangle) that can be put into the practice (Supplementary Figure 5). In the achievement of enlargement, this scope of action the mobilization of all intellectual forces is imperative. Because sport has served the emancipation of the “second sex” (Beauvoir, 1953), several studies have been focused on the importance of gender equality via gender quotas on sport boards (e.g., Knoppers and McLachlan, 2018; Piggott, 2021). Anti-doping sciences should and could demonstrate the possibility and importance of overcoming gender inequalities due to its high degree of inter- and multidisciplinarity, and show that gendered perspectives can have an impact on how the anti-doping research field is evolving (e.g., research priorities or interpretations of findings).

### Future Research Directions

In the analyses presented in this study, we have investigated diverse structural characteristics of women's involvement in anti-doping research. This diversity, however, triggered several further questions and promising research lines that should be addressed in future work. As a direct extension of this research, future directions should include the following research directions:

1. The scientific impact of women's contribution to anti-doping science. A topic of outstanding importance is the exploration of the impact of women-related anti-doping research on the scientific community. The impact of research in terms of citation indicators and measures, and also in bibliometric quality measures (such as journal metrics like the Journal Impact Factor, or the corresponding journal quartiles) reveals the extent of both the recognition and the scientific quality of women's contribution to anti-doping science. Recognizing that real research impact cannot be ascertained by journal metrics, future research is warranted to develop standardisable qualitative approaches for research impact assessment. These may include path analysis using bibliometric data and citations, exploring the mentions and uptake of the research findings outside academia, or a form of expert/peer-assessment or self-assessment of the research output based on its own merit (as opposed to the journal where it was published).
2. The key actors of knowledge integration in anti-doping science. An equally important topic is the identification of actors (authors, groups) within the anti-doping research landscape who plays the role of connecting different research fields in their work. By doing so, these actors facilitate knowledge integration in the multidisciplinary domain of anti-doping science and strengthen the evidence that women have a higher share in highly interdisciplinary research (Elsevier, 2021). Exploring the involvement of women in this process (via bibliometric science mapping tools used in modeling interdisciplinarity) can greatly improve our understanding of their potential in the field, which then can lead to tailored support for women in anti-doping research.
3. The analyses presented in this work applied the analytic framework of science mapping, the latter being a state-of-the-art toolbox of computational social science, but largely explorative in nature. A natural step is to complement these results with an explanatory approach in the sequel, with an investigation of how the structural features of women's involvement (such as authorship positions, research fields, institutional background, “invisible college” status, etc.) affect their success in the field (as measured, for example, via recognition measures such as citation measures). Finally, future investigations should use this study, and other bibliometric analyses, as a basis for follow-up studies, and explore the key results qualitatively, or via utilizing mixed-methodology.

## Conclusion

Monitoring gender representation in science, research, and technology is vital for progressing from under-representation of women to awareness, equity, and transformation in all subject areas, including anti-doping research. Gender inequalities in sport science in general, and in anti-doping research in particular, have been poorly recognized. With rapid advances in computational social science, modern bibliometric analyses now afford quantification of the potential differences and uncover the cognitive and social structure of anti-doping research in both hard- and social sciences. The comprehensive analysis of the academic publications in this study indicates that anti-doping research is a closed community with only 70 authors producing more than one output, and women have been under-represented in the field of anti-doping research for a long time, but their presence in and contribution to the field is evolving and steadily increasing. WADA's direct and indirect impact on research through funding and governance structure through the most influential men and women authors is noted and warrants further exploration. Anti-doping is in need to mobilize all intellectual capacities to tackle new challenges that threaten the integrity of elite sport, and women can play a unique role in this. The significantly higher information centrality of women means that they are better positioned to control and facilitate the information flow in the system, which can be highly beneficial in problem-focused multidisciplinary research as well as in fostering new collaborations both within the anti-doping community and with researchers in cognate fields. Bibliometric approach has the potential to play a significant role in anti-doping research. Alongside traditional systematic literature reviews and meta-analyses, bibliometric analyses can make a unique contribution to delineating the research field, map the knowledge base, and portray hidden relations in the field of anti-doping science.

## Data Availability Statement

The original contributions presented in the study are included in the article/Supplementary Material, further inquiries can be directed to the corresponding author/s.

## Author Contributions

AK, SS, and AP contributed to the conception and design of the study. SS organized the database. ZL performed the statistical analysis. AK and ZL wrote the first draft of the manuscript. SS, AP, and ZL wrote sections of the manuscript. AK, AP, and ZL contributed to writing, review, and editing of the manuscript. All authors contributed to manuscript revision, read, and approved the submitted version.

## Conflict of Interest

The authors declare that the research was conducted in the absence of any commercial or financial relationships that could be construed as a potential conflict of interest.

## Publisher's Note

All claims expressed in this article are solely those of the authors and do not necessarily represent those of their affiliated organizations, or those of the publisher, the editors and the reviewers. Any product that may be evaluated in this article, or claim that may be made by its manufacturer, is not guaranteed or endorsed by the publisher.
