# Supplementary material for: Women's Footprint in Anti-Doping Sciences: A Bibliometric Approach to Research Impact
Source: Front Sports Act Living. 2022 May 30;4:866648. doi: 10.3389/fspor.2022.866648 (PMC9195593; doi:10.3389/fspor.2022.866648)
Supplement: Supplementary file 1 [file Data_Sheet_1.DOCX]

Supplementary Material

Supplementary Figures


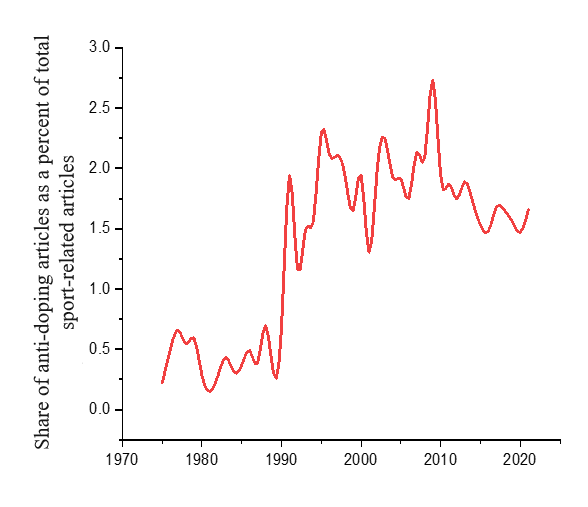


**Supplementary Figure 1**. Share of anti-doping articles from all sport-related articles


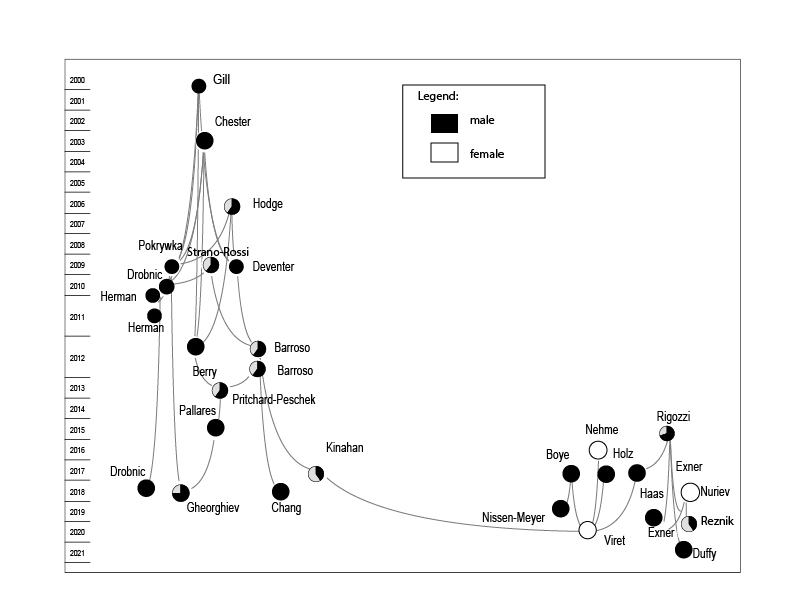


**Supplementary Figure 2**. Key publications in and women's contribution of the anti-doping control practice (No.5) cluster


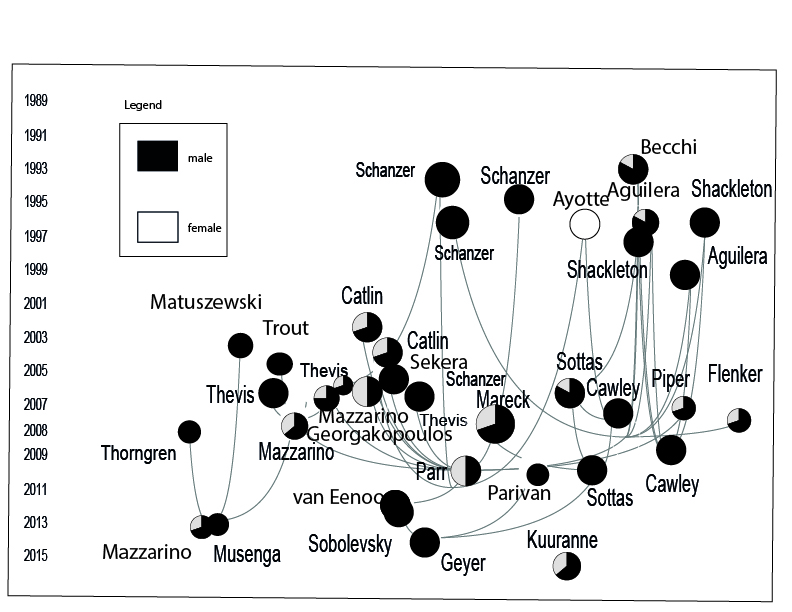


**Supplementary Figure 3**. Key publications in and women's contribution of the analytical method development cluster (the size of circles is proportional with citation of different sources)


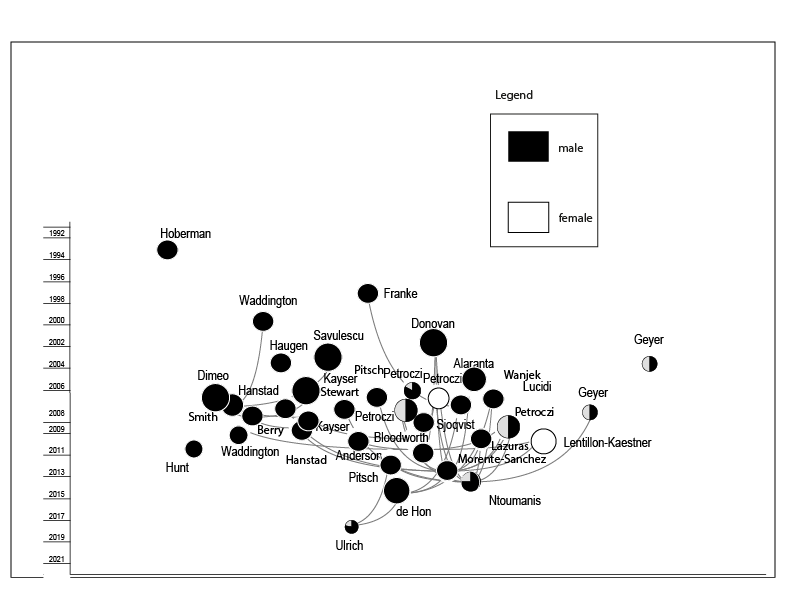


**Supplementary Figure 4**. Key publications in and women's contribution of the anti-doping policy development cluster (the size of circles is proportional with citation of different sources)


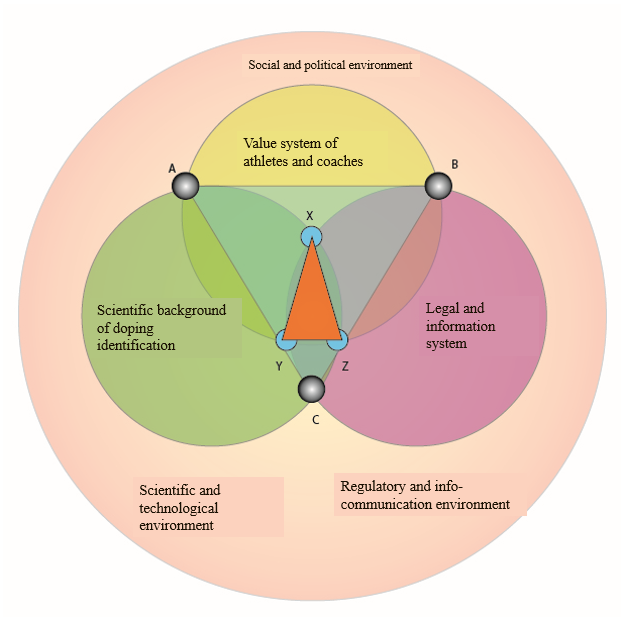


**Supplementary Figure 5.** Application of triple helix model to the anti-doping-related research and policymaking field
